# 

**Published:** 1889-08-10

**Authors:** 


SATURDAY, AUGUST 10th, 1889.

**Doctors as Policemen** - - - 287

**Words of Consolation**—The Love of Man to God - - 288

**Crime and Criminals** - - - 289

**Children's Country Holidays Fund** - - - 290

**The Student's Column.**—Edinburgh Medical Examinations 290

**The Doctor's Pulpit.**—In Nature's School - - 291

**Within the Wards.**—Bacterial Diarrhœa at Leicester—"Squint" and Spectacles - - 292

**The Story of the Insane from Year to Year.**—Asylum Reports - - 293

**Notes and News** - - - 294

**Everybody's Page** - - - 296

**Annotations.**—Elixirs of Life—A "Salvation Army" Hospital—Gipsy and Van Children - - 297

**Her Heart's Mistake.** By E. D. Cumming. Chap. xvii. 298

**Our Poorer Neighbours.**—How to provide for the Winter's Distress - - 299

**New Preparations.**—Antiseptic Dressings - - 301

**Amusements and Relaxation** - - - 302

**Scraps and Gleanings** - - - 302

**EXTRA SUPPLEMENT—THE NURSING MIRROR.**

**En Passant.**—Nurses as Witnesses—A Day's Pleasure—"Recollections of a Nurse"—A Foreign Visitor—Brutality—Nursing the Insane—Registration - - lxxi

**Physiology for Nurses.**—The Hair and Nails - - lxxii

**The Power of Laughter** - - - lxxii

**A Probationer's Diary** - - - lxxiii

**Winter Amusements and Remunerative Work.**—An Exhibition of Doll Nurses - - lxxiv

**Everybody's Opinion.**—Registration - - lxxiv

**Appointments—Examination Questions** - - lxxiv

**Vacancies** - - - lxxiv

**Notes and Queries** - - - lxxiv

